# Exploration of Feasible Immune Biomarkers for Immune Checkpoint Inhibitors in Head and Neck Squamous Cell Carcinoma Treatment in Real World Clinical Practice

**DOI:** 10.3390/ijms21207621

**Published:** 2020-10-15

**Authors:** Hui-Ching Wang, Tsung-Jang Yeh, Leong-Perng Chan, Chin-Mu Hsu, Shih-Feng Cho

**Affiliations:** 1 Graduate Institute of Clinical Medicine, College of Medicine, Kaohsiung Medical University, Kaohsiung 807, Taiwan; joellewang66@gmail.com (H.-C.W.); aw7719@gmail.com (T.-J.Y.); 930607@kmuh.org.tw (L.-P.C.); 2 Division of Hematology and Oncology, Department of Internal Medicine, Kaohsiung Medical University Hospital, Kaohsiung Medical University, Kaohsiung 807, Taiwan; e12013@gmail.com; 3 Drug Development and Value Creation Research Center, Kaohsiung Medical University, Kaohsiung 807, Taiwan; 4 Faculty of Medicine, College of Medicine, Kaohsiung Medical University, Kaohsiung 807, Taiwan; 5 Department of Otolaryngology-Head and Neck Surgery, Kaohsiung Medical University Hospital, Kaohsiung Medical University, Kaohsiung 807, Taiwan

**Keywords:** head and neck cancer, immunotherapy, immune checkpoint inhibitor, biomarker

## Abstract

Recurrent locally advanced or metastatic head and neck squamous cell carcinoma (HNSCC) is associated with dismal prognosis because of its highly invasive behavior and resistance to conventional intensive chemotherapy. The combination of targeted therapy and conventional chemotherapy has significantly improved clinical outcomes. In recent years, the development of immunotherapies, such as immune checkpoint inhibitors (ICIs), has further increased treatment responses and prolonged survival. However, the limited response rate, risk of immunotherapy-related adverse effects and high cost of immunotherapy make the identification of predictive markers to optimize treatment efficacy a critical issue. Biomarkers are biological molecules that have been widely utilized to predict treatment response to certain treatments and clinical outcomes or to detect disease. An ideal biomarker should exhibit good predictive ability, which can guide healthcare professionals to achieve optimal treatment goals and bring clinical benefit to patients. In this review, we summarized the results of recent and important studies focused on HNSCC ICI immunotherapy and discussed potential biomarkers including their strengths and limitations, aiming to gain more insight into HNSCC immunotherapy in real world clinical practice.

## 1. Introduction

Head and neck cancer, mainly head and neck squamous cell carcinoma (HNSCC) developing from the mucosa of the nasal and oral cavity, oropharynx, hypopharynx, or larynx, is the sixth most common cancer type globally [1]. Patients with early-stage HNSCC have satisfying outcomes after local treatment; however, the majority of HNSCC patients present with locally advanced disease at diagnosis [2]. Despite the incorporation of multimodal therapeutic modalities, including platinum-based chemoradiation, more than 50% of patients with locally advanced HNSCC experience recurrence or develop metastases (or both) within three years of treatment [3,4]. In recent years, the introduction of the targeted agent cetuximab targeting epidermal growth factor receptor has shown significant improvement in overall survival (OS) when combined with platinum-based chemotherapy [5,6]. However, there are still some unmet needs, and the overall response rate and survival remains suboptimal.

Immune checkpoint inhibitors (ICIs) are an important breakthrough in cancer treatment. ICIs targeting cytotoxic T lymphocyte antigen 4 (CTLA-4) and programmed cell death protein-1 (PD-1) and its ligand programmed death ligand-1 (PD-L1) [7] have demonstrated a significant and consistent benefit to survival when compared with standard treatments in prospective randomized clinical trials [8,9,10,11,12], leading to regulatory approval for several cancer types, including HNSCC. Importantly, the overall response rate to ICI monotherapy ranges from approximately 20% to 40%, suggesting that a substantial section of patients may not derive benefit from this treatment [13,14]. In addition, novel ICIs are costly and associated with potentially life-threatening immune-related effects [15,16,17]. With the rapid increase in approved indication and usage of ICIs in cancer treatment, how to achieve ideal treatment response, avoid toxicity, and reduce cost are emerging as important issues for related healthcare professionals in real world clinical practice.

To address the above issues, several efforts have been made to improve the efficacy of ICIs, such as combination with other immunotherapeutic agents or conventional chemotherapy. Another strategy is to identify feasible biomarkers that can help select suitable patients who may obtain the most benefit from ICI treatment in real world clinical practice. Currently, the ideal and robust predictive marker of ICI treatment response remains to be explored. In this review, we focus on key findings of important ICI clinical studies that include biomarker analysis. In addition, we also discuss emerging biomarkers and experimental models with predictive potential.

## 2. The Definition and Utilization of Biomarkers

Based on the definition created by the U.S. Food and Drug Administration (FDA) and the National Institutes of Health (NIH), a biomarker is a defined characteristic that can be used as a measureable indicator of normal biological condition, disease processes or responses to exposure or intervention [18]. With different applications, there are several subtypes of biomarkers. In addition, a single biomarker can be used to meet multiple criteria for different uses if the evidence is developed. Currently, biomarkers include diagnostic, monitoring, predictive, pharmacodynamic/response, and prognostic biomarkers.

Regarding cancer treatment, biomarkers play several important roles. For example, biomarkers can assist in the diagnosis of cancer (diagnostic role), indicate possible clinical outcomes (prognostic role), improve patient selection for a specific treatment or enrolment in clinical trials (predictive role), and define the most effective dosage of therapeutic agents (pharmacodynamic role) [19].

Based on the above description, an ideal biomarker for treatment prediction should exhibit high predictive ability for clinically meaningful benefit. Moreover, the analysis and detection tool of biomarkers should also be as commonly available and cost-effective as possible to make implantation and application of the markers clinically significant in real world setting [20].

## 3. Biomarkers in HNSCC Treatment in the Conventional Treatment Era

Several important tumor markers have been identified as potential biomarkers in HNSCC since these markers have been confirmed or validated in several clinical studies [21]. Some markers are associated with a better response to chemotherapy or concurrent chemoradiation and better survival, while others are not. To date, at least seventy markers have been evaluated and reported. Among these markers, some have shown evidence as prognostic markers when the expression level was evaluated in clinical trials, including epidermal growth factor receptor [22,23,24], p16 [25,26,27], human papillomavirus (HPV) [27,28,29], cyclin D1 (CCND1) [30,31], B cell lymphoma-extra large (Bcl-xL)/Bcl-2 [32,33] and ERCC1 [34,35]. In addition, the amplification of genes such as EMS1 [36], FGFR1 [37], and CCND1 [38] is also related to clinical outcome. Moreover, a recent study performed by computational analysis revealed the mutational profile of TP53 would be a predictive factor for prognosis [39].

## 4. Potential Biomarkers in HNSCC Immunotherapy

**Figure 1:**
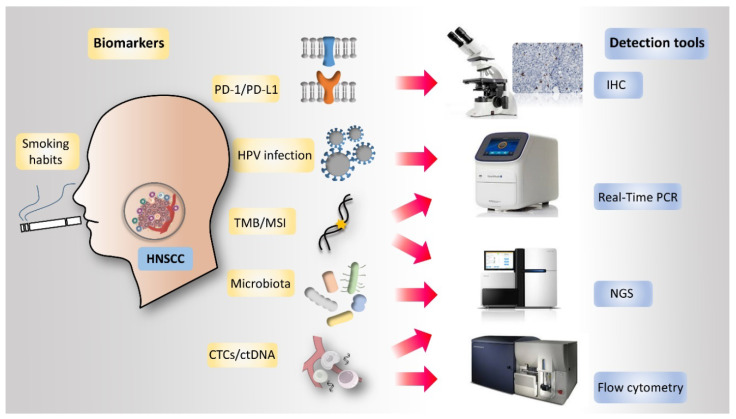
Current and emerging biomarkers for prediction of the clinical efficacy of immune checkpoint inhibitors (ICIs) in head and neck squamous cell carcinoma (HNSCC). Several host- or tumor-related markers have been demonstrated to be able to predict the clinical efficacy of ICI treatment. Advances in molecular analysis have also provided valuable predictive information such as tumor mutational burden (TMB) and status of microsatellite instability (MSI). Other markers, including circulating tumor cells (CTCs), circulating tumor DNA (ctDNA), and gut or oral cavity microbiota are also being investigated. CTC, circulating tumor cells; ctDNA, circulating tumor DNA; HNSCC, head and neck squamous cell carcinoma; ICIs, immune checkpoint inhibitors; MSI, microsatellite instability; TMB, tumor mutational burden.

Studies exploring molecular biomarkers in HNSCC treatment have been performed for many years, but there is still no consensus for clinical practice. Recent studies have shown that some biomarkers may exhibit significant potential to guide treatment decision making. These emerging biomarkers include PD-L1 expression on cancer cells, human papillomavirus (HPV) infection status, tumor mutational burden (TMB), tumor immune infiltration, T cell-inflamed gene expression profile (GEP), smoking history, microsatellite instability (MSI), circulating tumor cells (CTCs) and circulating tumor DNA (ctDNA) (Figure 1). In recent years, the intestinal microbiota has been found to play a role in the modulation of host anticancer immune responses and alter the anticancer effect of chemotherapy or immunotherapy [40,41]. Currently, great efforts are being made to identify novel and reliable markers, as well as further verification of some markers that have shown potential in the previous studies, aiming to determine a biomarker to guide HNSCC immunotherapy in real world setting.

## 5. PD-L1 Expression

The PD-1/PD-L1 axis plays a critical role in the magnitude of the inflammatory response and maintains immune homeostasis. PD-L1 is expressed on various normal and immune cells in the tumor microenvironment and is much more commonly present than PD-L2 [42]. PD-1, mainly expressed on the surface of activated T and B cells, maintains peripheral and central immune cell tolerance by binding to its ligands, PD-L1 and PD-L2, and inhibiting the activation of peripheral T cells [43]. In the tumor microenvironment (TME), tumor cells can utilize the PD-1/PD-L1 axis to suppress immune surveillance and promote their own growth [44]. In HNSCC, higher PD-L1 expression is associated with advanced disease status and poorer prognosis [45,46]. Higher PD-L1 expression in lung metastatic tumors was also found to be associated with poor outcome in recurrent and metastatic HNSCC (R/M HNSCC) patients after complete metastasectomy [47]. In the era of immunotherapy, ICIs, including PD-1/PD-L1 inhibitors, can block negative regulatory signaling pathways, leading to activation of T cells from an exhausted status, and then promote subsequent T cell-mediated cancer cell killing.

With respect to biomarkers for ICI treatment, PD-L1 is one of the most common markers under clinical investigation (Table 1). Currently, PD-L1 expression is determined by immunohistochemistry. The status of PD-L1 expression (positive or negative) is measured by calculating the proportion of PD-L1-expressing tumor cells and/or immune cells. Theoretically, tumor cells with PD-L1 expression tend to be more sensitive to PD-1/PD-L1 blockade treatment than PD-L1-negative tumor cells [48]. These correlations were also observed in some clinical studies with different PD-1/PD-L1 inhibitors across various tumor types [10,49]. In R/M HNSCC trials, a higher response rate and better survival in patients with high PD-L1 expression were also observed in the subgroup analysis of some ICI trials, including KEYNOTE-040 and KEYNOTE-048 [9,50,51]. In these two studies, a combined positive score (CPS) incorporated PD-L1-positive tumor and immune cells to define PD-L1 positivity. In the KEYNOTE-040 trial comparing the clinical efficacy of second-line pembrolizumab vs. investigators’ choice of standard of care (SOC) treatment, pembrolizumab showed superior OS (8.4 vs. 9.9 months; *p* = 0.0161) over SOC treatment. In the subgroup analysis, a survival benefit was observed in patients with a CPS ≥ 1. Among patients with CPS < 1, there was no obvious difference between the pembrolizumab and SOC groups [51]. In the KEYNOTE-048 trial, pembrolizumab alone or a pembrolizumab and chemotherapy combination were compared with the EXTREME regimen. Two cut-off values for the CPS were used for analysis (1 and 20). This study demonstrated that pembrolizumab treatment had better OS than the EXTREME regimen in patients with CPS ≥ 1 or CPS ≥ 20 [9].

In addition to the CPS, KEYNOTE-040 also used a tumor proportion score (TPS) to define PD-L1 expression on only tumor cells. The analysis revealed that a high value (TPS ≥ 50%) was significantly correlated with better clinical outcome, concordant with the findings in non-small-cell lung cancer in the KEYNOTE-010 study [52]. In the phase II HAWK study, durvalumab monotherapy showed antitumor activity in R/M HNSCC patients with higher PD-L1 expression (≥25%) [53]. In the CHECKMATE-141 trial, PD-L1 expression (cut-off values: 1%, 5%, and 10%) was also determined on only tumor cells. The patients who received nivolumab treatment had a significantly prolonged OS, including patients with PD-L1-negative HNSCC. Subgroup analysis showed that patients with PD-L1 expression ≥ 1% had a better median OS (8.7 vs. 4.6 months, HR: 0.36–0.83) than patients who received standard therapy, but the significance was not observed across all cut-off values [11,54].

The above results show a significant trend for higher PD-L1 expression being associated with more obvious clinical benefit. However, there are some issues to be addressed to further optimize anti-PD-1/PD-L1 treatment in clinical practice. For example, the assay for PD-L1 expression evaluation and the threshold to define positivity have not been standardized, making the launch of harmonization projects an urgent need. Second, the expression of PD-L1 on tumors is regulated by multiple molecular pathways that are altered in HNSCC [55,56]. Moreover, previous chemotherapy can affect the expression level of PD-L1 [57]. Hence, the expression of PD-L1 could be dynamic, changing from initial diagnosis to disease recurrence or progression, and may differ between primary and metastatic lesions [47,58,59]. Third, PD-L1 is expressed in both HNSCC cells and their surrounding immune cells, including regulatory T cells (Tregs), natural killer cells and antigen-presenting cells [60,61]. It remains controversial whether the expression of PD-L1 should consider all cells with PD-L1 expression or only PD-L1-expressing cancer cells. As mentioned above, the CPS demonstrated a positive association with treatment response and survival in the KEYNOTE-040 and KEYNOTE-048 trials [9,51]. When the positivity of PD-L1 expression on only tumor cells was considered, the KEYNOTE-040 study using the TPS demonstrated that a higher cut-off value for TPS (≥50%) was also linked to a significantly better clinical outcome [51]. In the CHECKMATE-141 study, different cut-off values (1%, 5%, and 10%) of PD-L1 expression positivity were used. Higher expression levels of PD-L1 were correlated with a better overall response rate in the PD-L1-positive group than in the standard therapy group. Regarding OS, patients with PD-L1 expression ≥1% and ≥5% had significantly better outcomes than patients in the standard therapy group. However, there was no positive correlation when higher cut-off values (≥10%) were used [11]. The difference in methodology suggests an urgent need to establish a consentient guideline for determining PD-L1 expression positivity for real-world R/M HNSCC ICI treatment. Recently, a comment from the Society for Immunotherapy of Cancer indicated that tumor PD-L1 expression is generally correlated with a better response in R/M HNSCC patients who receive anti-PD-1/PD-L1 ICI treatment. The predictive value can be further improved if PD-L1 expression is on tumor-infiltrating lymphocytes (such as in the CPS) [62]. Moreover, further validation and standardization of PD-L1 immunohistochemistry staining protocols to minimize interassay discrepancies are also ongoing [63,64,65].

## 6. HPV Infection Status

HPV infection status plays a critical role in the immunomodulation of HNSCC. Generally, HPV-positive HNSCCs demonstrate relatively inflamed immune environments compared with HPV-negative HNSCCs [66,67,68]. Compared with HPV-negative patients, HPV-positive oropharyngeal cancer has a relatively less immunosuppressive TME, as evidenced by a higher CD4+ cell count, higher CD8+ cell count, lower number of Tregs, higher PD-1 mRNA level, and lower CD4+/CD8+ ratio than the respective levels in HPV-negative HNSCC [67,69]. These immune profile distinctions may contribute to the response of the host immune system to viral or tumor antigens, leading to PD-L1 expression on immune cells. In a retrospective study analyzing 402 patients with resected HNSCC (mainly in the oral cavity and oropharynx), PD-L1 expression was evaluated on both tumor and immune cells. This study demonstrated that high PD-L1 expression (≥5%) on immune cells and high abundance of PD-1+ T cells and Foxp3+ Tregs were associated with better clinical outcome [70]. In another study using The Cancer Genome Atlas (TCGA) data from 280 HNSCC patients, the results of transcriptomic analysis show that HPV-positive tumors demonstrated higher immunogenicity than HPV-negative tumors, as evidenced by their larger infiltration of activated CD8+ T cells. HPV status did not affect PD-1 and PD-L1 expression. HPV+ tumors had higher CTLA-4 expression and Treg infiltration and a higher Treg/CD8 T cell ratio than HPV-negative tumors [71].

Taken together, these findings suggest that HPV-positive HNSCC patients may have a better response than HPV-negative patients when receiving ICIs. This hypothesis is first supported by the subgroup analysis of the pembrolizumab KEYNOTE-012 study, which showed a higher response rate in HPV-positive patients than HPV-negative patients (32% vs. 14%) [72]. However, these results were not observed in the following KEYNOTE-040 and KEYNOTE-055 trials or the CHECKMATE-0141 trials, which used nivolumab. In KEYNOTE-040, which used p16 as a marker of HPV infection, p16+ patients did not have a better OS than p16- patients [51]. In the KEYNOTE-055 study, there was also no significant difference in OS between HPV-positive and HPV-negative patients (16% vs. 15%) [73]. Other ICI trials using PD-L1 inhibitors also showed mixed results. For example, a higher response rate was observed in HPV+ patients treated with durvalumab than in HPV-negative patients treated with durvalumab (29.4% vs. 10.8%), while no difference was observed in the atezolizumab trial (15% vs. 17%) [53,74]. A recent pooled analysis of data from six clinical trials investigated the efficacy of a PD-1/PD-L1 inhibitor in HPV+ and HPV- HNSCC patients and revealed that HPV+ patients benefited more from PD-1/PD-L1 inhibitors than HPV- patients, including better responses and survival (OS: hazard ratio = 0.71, *p* = 0.02, overall response rate: 21.9% vs. 14.1%, odds ratio (OR) = 1.79, *p* = 0.01) [68]. The inconsistent findings of the above studies suggest that HPV infection status may not be the only predictive marker, other factors, such as PD-L1 expression, tumor mutational burden, and immune infiltration, should be taken into consideration.

Apolipoprotein-B mRNA editing enzyme catalytic polypeptide-like (APOBEC) enzymes, a family that catalyzes the deamination of cytosine bases, have been linked to mutagenesis in HPV-positive HNSCC [75]. HPV-positive HNSCC tumors exhibited a higher APOBEC signature than HPV-negative tumors [76]. In addition, high APOBEC activity was correlated with upregulated immune signaling pathways, which might be linked to better ICI sensitivity [77]. A recent study evaluating whole-exome and RNA sequencing data from the TCGA dataset demonstrated that the APOBEC mutational burden was closely correlated with tumor-specific neoantigens, a marker suggesting a better response to ICI immunotherapy [78,79]. However, the predictive role of the APOBEC mutational burden in ICI treatment of HNSCC requires further study.

## 7. Tumor Mutational Burden (TMB)

TMB, which analyzes the number of somatic mutations per DNA megabase (Mb), has been investigated as a potential predictive marker in ICI immunotherapy. Currently, TMB is regarded as a promising predictive biomarker of responsiveness to ICIs across 27 tumor types and subtypes according to a retrospective analysis [80].

Theoretically, a higher missense mutation number is correlated with a higher number of tumor neoantigens, which may induce a more significant immune response and increase the response to ICI treatment. In non-small-cell lung cancer patients who received nivolumab treatment, a higher mutational burden (≥10 mutations per Mb) was found to be associated with better progression-free survival (PFS), regardless of PD-L1 expression level [81]. In HNSCC, combined analysis of ICI pembrolizumab trials revealed that the TMB, the CPS, and an inflamed GEP were three major parameters associated with the best overall response, regardless of HPV infection status. Additionally, there was no significant correlation between TMB and GEP or PD-L1. As HPV status was not stratified in this analysis, the findings suggest that TMB and inflammatory biomarkers may demonstrate different and independent predictive values [82,83].

Another study evaluated 126 HNSCC patients and showed that ICI responders had significantly higher TMB levels than nonresponders (21.3 vs. 8.2 mutations/Mb). HNSCC patients with TMB ≥ 10 mutations/Mb had a longer median survival than patients with TMB ≥ 5 mutations/Mb (20.0 versus 6.0 months, *p* = 0.01). The subgroup analysis revealed that virus-positive (HPV-positive/Epstein-Barr virus (EBV)-positive) patients had a lower TMB than virus-negative patients but an improved OS. However, TMB status was not correlated with survival in the virus-positive patients. In virus-negative patients, a higher TMB (≥10 mutations/Mb) was associated with better survival. Interestingly, smokers had higher TMB levels than nonsmokers (10.3 versus 5.3, *p* = 0.01) in both the HPV-negative and HPV-positive groups. However, the response to ICIs could not be predicted according to smoking status in the multivariate analysis (*p* = 0.62) [84].

A randomized, open-label, phase three EAGLE trial (NCT02369874) evaluated plasma-based tumor mutational burden (bTMB) as a predictor for survival in 247 R/M HNSCC patients [85]. OS and PFS were significantly improved for immunotherapy (monotherapy or combined therapy) vs. chemotherapy in patients with high bTMB (≥16 mut/Mb) vs. low (<16 mut/Mb). Patients with higher bTMB gained more benefits in immunotherapy compared with chemotherapy. Further validation of bTMB as a predictive biomarker is ongoing.

## 8. Tumor Immune Infiltration

The TME is characterized by heterogeneous molecular and cellular components, as well as complex interactions between tumor cells and surrounding immune cells. Multiple immune cells coexist with tumor cells in the TME, including tumor-infiltrating lymphocytes (TILs, CD8+ T cells, Tregs, B cells), natural killer (NK) cells, macrophages, antigen-presenting cells (APCs), and myeloid-derived suppressive cells [66]. In a recent study investigating oropharyngeal squamous cell carcinoma (SCC) patients, HPV+ tumors had significantly higher densities of CD20+ B cells and CD8+ T cells than HPV- tumors. Importantly, tumors with high B cell infiltration density showed significantly reduced immunosuppressive regulatory B cells. A high density of tumor-infiltrating B cells and significant direct B cell/CD8+ T cell interactions were related to good prognosis [86].

With respect to the investigation of predictive markers for immunotherapy, a study retrospectively evaluating R/M HNSCC patients treated with anti-PD-1/PD-L1 ICI treatment showed that increased intratumoral CD8+ T cell infiltration and an increased CD8+ T cell/Treg ratio were linked to a better treatment response [84]. The application of the immunoscore (IS), which quantifies the density of intratumoral CD8+ cells at the tumor margin, has also been analyzed in other studies. A higher IS has been observed to be related to better long-term survival in early-stage colon cancer, melanoma and lung cancer [87,88,89]. In HNSCC, a higher IS is associated with better OS in patients with resectable HNSCC who undergo complete tumor removal [90]. Another study revealed that a high IS was associated with higher CD8+ T cells, lower CD4+ T cells, and higher MHC type 1 expression in tumor cells. Currently, the role of the IS in the prediction of ICI efficacy is yet to be determined [91].

Incorporation of other immune checkpoint molecules into an analysis is another way to provide more information. The expression of T cell immunoglobulin and mucin domain-containing protein 3 (TIM-3), T cell immunoreceptor with Ig and lymphocyte-activating gene 3 (LAG-3) and ITIM domains (TIGIT) on T cells is associated with impaired T cell immune response [92]. In melanoma and non-small-cell lung cancer clinical trials, the expression of the above molecules was associated with resistance to ICI [93,94,95,96]. In HNSCC, nonresponders to ICI treatment have more significant intratumoral infiltration of exhausted PD-1+ CD8+ cells with TIM-3 and LAG-3 expression than patients who respond [84]. A subgroup analysis of the CHECKMATE-141 trial evaluated nivolumab treatment beyond disease progression and showed that responders had PD1+ Tregs numbers on day 43 of treatment that were significantly lower than baseline levels and the levels in nonresponders, suggesting that circulating exhausted T cells could be a predictor of ICI treatment [97].

## 9. T Cell-Inflamed Gene Expression Profile (GEP)

Recent studies have shown that the gene expression profile (GEP) of tumors provides valuable information for predicting treatment response and prognosis. For example, tumors with an inflamed phenotype were shown to be more sensitive to anti-PD-1/PD-L1 agents than those without an inflamed phenotype [98]. A study evaluating samples from non-small-cell lung cancer, HNSCC and melanoma demonstrated that PD-1 and PD-L1 expression, together with 11 signatures including CD8+ and CD4+ T cell activation, NK cells, and interferon (IFN) activation, were associated with better disease control and PFS [99]. Another study using data from TCGA, GSE40774 and MSK-IMPACT, as well as data from six clinical trials, revealed that HPV was a predictive biomarker regardless of PD-L1 expression. HPV-positive patients had higher cytolytic activity than HPV-negative patients. In addition, the IFN-γ-related gene signatures were closely related to HPV-positive status, whereas the immunosuppressive IL6/TGF-β-related gene signatures were related to HPV-negative status. However, the multivariate analysis to investigate prognostic factors revealed that some immune-related genes (CD8A, CD4, TGFB1 and CTLA4) were independent factors, but HPV status was not [68]. In the subgroup analysis of KEYNOTE-012, all six IFNγ-related genes (CXCL9, CXCL10, IDO1, IFNG, HLA-DRA, and STAT1) had significantly higher mean expression values in responders than in nonresponder patients. The Youden index threshold incorporating the above genes showed a 95% negative predictive value, which may help exclude potential nonresponders before pembrolizumab treatment [100].

## 10. Smoking Status

Smoking is one of the major risk factors for the development of HNSCC and can have pro-inflammatory and immunosuppressive effects on the TME, leading to the growth of tumor cells [101]. In the era of HNSCC immunotherapy, smoking is known to induce DNA damage and genetic mutations, causing higher overall mutational loads and enriched immunogenic neoantigens. However, these effects were overcome by a profoundly immunosuppressive microenvironment [102,103]. An analysis from sequencing data of HNSCC samples revealed that a high mutational smoking signature was associated with lower levels of immune infiltration, cytolytic activity, and IFNγ pathway signaling than a low signature. Importantly, several immune-related genes were downregulated in HNSCC patients with the heaviest tobacco usage, including T cell receptors, immunoregulatory molecules, cytotoxic effectors, cytokines, and MHCII molecules [102].

The effect of smoking on the outcome of immunotherapy has been evaluated in some clinical studies. In the subgroup analysis of the CHECKMATE-141 study, smokers trended towards having an inferior clinical outcome compared with nonsmokers [11]. In another retrospective study analyzing 81 HNSCC patients treated with PD-1 or PD-L1 inhibitors, a significantly poorer outcome was observed in HPV-negative former or current smokers than in those who never smoked [102].

## 11. Microsatellite Instability (MSI)

MSI is a genomic condition of the tandem repeats hypermutability, determined by capillary electrophoresis or next-generation sequencing platforms, which have been investigated as optimal markers for biological phenotypes and clinical outcomes in HNSCC. MSI, in particular, has been associated with the response of active immune checkpoint blockade in cancer therapy and related with impaired DNA mismatch repair. Previous studies have suggested that tumors with more mutations affecting the DNA damage response, such as high microsatellite instability (MSI-H) or mismatch repair deficiency (dMMR) tumors, were associated with higher TMB and were more sensitive to ICIs than tumors with mutations affecting other pathways, leading to FDA approval of ICI treatment (pembrolizumab) for patients with dMMR or MSI-H tumors regardless of histology [80,104,105,106]. In HNSCC, a retrospective analysis showed that high MSI was related to a durable response from ICI treatment [107]. Currently, recommendations for MSI testing for immunotherapy are under development [108].

## 12. Circulating Tumor Cells (CTCs) and Circulating Tumor DNA (ctDNA)

CTCs are rare epithelial cells that outflow from the primary tumor to the bloodstream, which can be extracted via the liquid biopsy method and we can analyze the genetic alternations at the level of DNA, RNA and protein [109,110,111]. In addition to CTCs, ctDNA is another material with 150–200 bp fragment DNA released from the tumor cells undergoing apoptosis or necrosis into the blood [111,112,113]. Compared with tissue biopsy, which remains the gold standard in the diagnosis of solid malignancies, analysis of CTCs or ctDNA are emerging as an important diagnostic tool because of it being time-saving, non-invasive, cheaper and there is lower risk of cancer spreading [113,114,115,116]. In HNSCC, investigation of CTCs and ctDNA in different disease or treatment status has been considered as a predictive marker for diagnosis, prognosis and response to treatment [55,109,110,114,117,118,119]. Additionally, positive CTCs after major treatment are associated with an increased risk of distal metastasis in patients with localized HNSCC [120,121].

In the immunotherapy era, combined analysis of PD-L1 and CTC show mixed results in terms of clinical outcome. For example, a study revealed that patients with HNSCC had shorter PFS and OS when overexpressed PD-L1 was observed in the epithelial cell adhesion molecule (EpCAM)+ CTC after treatment [120]. Another study demonstrated the clinical outcome of HNSCC patients was significantly associated with CTC number and circulating cancer stem-like cells (cCSCs) ratio, but not PD-1 expression on peripheral CD4+, CD8+, or CD56+ cells [122]. Regarding response to ICI treatment, some small scale studies that analyzed PD-L1 status in CTCs show the potential to predict post-ICI treatment outcomes in patients with metastatic lung cancer [123,124,125]. In the ctDNA study, the level of baseline ctDNA was correlated with the OS and PFS when cancer patients were (including HNSCC) treated with pembrolizumab. The degree of ctDNA reduction after pembrolizumab treatment, independently of PD-L1 expression, was closely associated with prognosis [113]. In summary, more studies are still need to confirm the predictive role of CTC and ctDNA in HNSCC treated with ICIs.

## 13. Microbiota

In recent years, accumulating evidence has suggested that the intestinal microbiota can regulate the anticancer response of the host and the response to anticancer treatment, including immunotherapy [126,127,128,129,130]. In HNSCC, tumors tend to develop from the epithelium and mucosa of the oral cavity and pharynx, and both sites are consistently exposed to various factors from the outside environment, leading to alteration of the oral microbiota [131]. A study evaluating the saliva of HNSCC patients and healthy individuals revealed distinct microbiota compositions, and the presence of certain bacteria was related to a lower risk of HNSCC [132,133]. A recent study analyzing normal, primary tumor, and metastatic HNSCC tumor areas demonstrated the relative abundance of *Fusobacterium* in primary and metastatic cancer tissues, whereas the abundance of *Streptococcus* was significantly decreased [134].

The role of the microbiota in the prediction of ICI treatment efficacy in HNSCC patients has also yet to be determined. The data so far have only been from subgroup analyses of the CHECKMATE-141 study, which evaluated the oral microbiota in saliva from HNSCC patients treated with nivolumab; however, no significant correlation with treatment response or survival was observed [135].

## 14. Organoids: A New Ex Vivo Experimental Model for Biomarker Study

Organoids is a novel three-dimensional cultural system generated from fresh tissue samples from human tumors. These cultural models can be established without time-consuming ex vivo selection and are faster, simpler, and less costly to generate than patient-derived xenograft mouse models. Compared with the conventional two-dimensional cultural system, organoids can mimic the natural microenvironment, which allow further study of vascularization and blood perfusion [136,137]. Emerging evidence has revealed that organoids can be used as a model system of tumor-immune microenvironment for prediction of anti-tumor drug efficiency [138,139]. In a recent study, patient derived organoids collected from biopsy tissue of chordoma were used to evaluate the efficacy of anti-PD 1 agent nivolumab. This study successfully demonstrated heterogeneous distribution of PD-L1+ cells (determined by immunohistochemistry) in the tumor tissues. In addition, the response to nivolumab was also investigated by using this model [140].

Regarding organoids in HNSCC research, a recent study showed a rapid outgrowth of HNSCC tumor organoids with high efficiency, which can serve as a platform for investigations including tumor phenotypes, drug resistance, synergistic effect of combined therapy, and identification of effective target therapies [141]. We can expect more studies using organoids to evaluate response of ICIs in HNSCC to be reported in the near future.

## 15. Perspectives and Conclusions

Immunotherapy has revolutionized the treatment landscape for R/M HNSCC, demonstrating clinical benefits including prolonged disease control and survival in some patients. With increasing usage of this therapeutic modality, how to further optimize the efficacy is an important issue. The development of suitable predictive biomarkers may provide valuable information for treatment decision making. The summary of biomarkers with supporting clinical data is listed in Table 2.

PD-L1 expression on tumor cells is the most widely used biomarker in clinical practice. It is likely that using higher cut-off value of PD-L1 positivity can identify the group that may truly get benefit from ICI immunotherapy. However, there are some limitations, making it an imperfect marker. Currently, there are four immunohistochemical assays for specific ICIs (PD-1/PD-L1 inhibitors). Different assays and different cut-off values for PD-L1 positivity in different clinical studies have shown inconsistent results. Harmonization of assays, methodologies, and cut-off values may provide valuable information to address the above issues. However, there are still some HNSCC patients with negative PD-L1 expression who are responsive to ICI treatment [11,74], suggesting that the inclusion of other predictive markers is a possible solution. A combined analysis including PD-L1 expression and tumor-infiltrating lymphocytes, as well as the T cell phenotype, has shown promising results [84,97]. Since the available data are from retrospective analyses, they may provide a rationale for further prospective clinical investigations to evaluate the clinical efficacy of biomarker-guided treatment.

Other parameters or technologies providing predictive information for ICI treatment include the TMB, MSI, and GEPs. These parameters all showed predictive potential for ICI treatment. In a recent study that systematically analyzed various parameters, CD8+ T-cell abundance, TMB, and high PD1 gene expression were most predictive factor for ICI treatment across several tumor types including HNSCC [142]. However, the investigation of TMB or GEPs requires advanced molecular examination technology, which is highly complex and costly, making their use in clinical practice difficult. MSI or dMMR status, another parameter suggesting high TMB, has been shown to qualify patients for pembrolizumab treatment. Regarding the microbiota, CTC, and ctDNA, more data are still needed to determine their role in ICI treatment in HNSCC patients. In term of a new experimental model for biomarker exploration, analysis by using organoids may provide more information. The detection methods, strengths, and limitations of each biomarker are listed in Table 3.

Taken together, these findings show that selecting patients for ICI immunotherapy merely based on a single parameter without taking other factors into consideration would be insufficient because HNSCC and its tumor microenvironment are highly heterogeneous. With the advancement of several diagnostic tools and molecular exams, we can expect that there will be increasing data available for interpretation and analysis. In the future, rapid development of artificial intelligence technologies with the capability to process massive amounts of data and analyze them may further revolutionize health care and help physicians predict clinical outcomes more accurately than they can with conventional statistical tools [143,144].

## Figures and Tables

**Table 1:** Evidence for programmed death ligand-1 (PD-L1) expression cut-off values and corresponding immunotherapeutic agents in clinical trials.

Trialsn (ICI, line)	Control	IHC Assay	PD-L1 Determination and the Cut-Off Values	Findings
CHECKMATE-141 (Nivolumab, 2nd)	Standard therapy	28-8	TC ≥ 1%, 5%, and 10% * (TC, tumor cells)	ORR Nivolumab vs. SOC: 13.3% vs. 5.8% OS (≥1%, 5%, and 10%): 8.7, 8.8, and 8.7 months
KEYNOTE-048 (Pembrolizumab, 1st)	EXTREME	22C3	CPS ≥ 20 or ≥ 1 (number of PD-L1+ cells (tumor cells, lymphocytes, and macrophages)/total number of tumor cells × 100)	Median OS (SOC:10.7 months) CPS ≥ 20: 14.7 months # CPS ≥ 1: 13.6 months #
KEYNOTE-040 (Pembrolizumab, 2nd)	SOC	22C3	TPS ≥ 50% (TPS, tumor proportion score = % of PD-L1+ tumor cells)	OS: 11.6 vs. 6.6 months
			CPS ≥ 1	OS: 8.7 vs. 7.1 months
HAWK (Durvalumab, 2nd)	- (single arm)	SP263	TC ≥ 25%	HPV+ vs. HPV- OS: 10.2 vs. 5.0 months

* A positive trend in clinical benefit was not observed when using higher cut-off values; # pembrolizumab combined with chemotherapy; CPS, combined positive score; ICI, immune checkpoint inhibitor; IHC, immunohistochemistry, ORR, overall response rate; OS, overall survival; PFS, progression-free survival; SOC, standard of care.

**Table 2:** Summary of biomarkers that have the potential to predict the clinical efficacy of immune checkpoint inhibitors in head and neck cancer.

Factors		Better Response	Poorer Response
Tumor-related	PDL-1	High	Low
	TMB	High	Low
	MSI	High	Low
TME-related	GEP	Inflamed	Noninflamed
	Immune profile	1.↑Intratumoral CD8+ T cell infiltration 2.↑CD8+ T cells/Tregs 3. Lower PD1+ Tregs (baseline) 4. Decreased PD-1+ CD8+ T cells (after treatment)	1.↑Exhausted PD-1+ CD8+ cells (TIM-3+ or LAG-3+)
Host-related	HPV status	HPV positive	HPV negative
	Smoking status	No	Yes

GEP, gene expression profile; HPV, human papillomavirus; MSI, microsatellite instability; TMB, tumor mutational burden; TME, tumor microenvironment.

**Table 3:** The present diagnostic biomarkers in HNSCC: detection methods/technique, strengths, and limitations.

Markers	Detection Tools	Gene/Protein	Methods	Strengths	Limitations	References
PD-1/PD-L1	IHC stain	PD-1 or PD-L1 protein expression	Analysis of the expression level of PD-1/PD-L1 in stained tissue slides	1. Many studies support. 2. Popular and relatively cost-effective detection tool.	1. Cell types detection need to be defined: tumor cell only/tumor cell+ immune cell/immune cells only 2. Ambiguous or inconsistency threshold or cut-off value	[12,47,49,50,51,52,53,61,62,63,64]
HPV	1. HPV viral titer 2. P16 IHC stain 3. HPV DNA In-Situ Hybridization 4. HPV RNA RT-PCR	1. HPV L1 region (GP5+/GP6+) 2. HPV 16E6/E7	1. IHC stain for P16 expression. 2. DNA L1 region (GP5+/GP6+) and genotype assay performed by PCR and HPV probes hybridization. 3. Amplification of 16E6/E7 mRNA.	1. HPV+ has anti-tumor immunity TME (higher immune-related cells: CD3+/CD4+/CD8+ T cells, CD45+ lymphocytes, CD19+/CD20+ B cells, CD56dim NK cells, APCs, DCs; lower number cells: Tregs cells; higher PD-1 mRNA expression; higher chemokines)	Mixed study results	[65,66,67,68]
MSI	The variation of tandem repeat sequences/MSI detection	MMR (MLH1, MSH2, MSH6 and PMS2) related genes and repeat sequences abundance regions	PCR followed by capillary electrophoresis or sequencing	1. Lower technology threshold 2. Few microsatellite markers can detect 3. Easy diagnosis than TMB 4. Correlated with the TMB	1. Prefer monophonic microsatellite 2. MSI accumulation in invasive carcinomas than precursor lesions	[104,105,106,107]
TMB	Detect the mutation rate in genes or genome	Whole exome, whole genome or selected genes	Analysis the mutations in the DNA level by NGS	1. Good predictive ability 2. Higher TMB is associated with better PFS and response	1. Limitation of data, not associated with GEP or PD-L1 2. Expensive and higher technology/analysis threshold	[79,80,81,82,83,107]
CTCs and ctDNA	CTCs separation or ctDNA isolation from peripheral blood	Whole genome or target genes analysis 1. Genes mutations detection in CTCs and ctDNA 2. mRNA expression in CTCs (PD-L1 mRNA expression in EpCAM+ CTCs) 3. Protein-expression	1. Microfluidic methods, immune-magnetic, and flow cytometry for CTCs collection 2. The CellSearch system approved by the FDA for CTC detection 3. ctDNA isolated from plasma with collection column.	1. Time-saving, noninvasive, and decrease cancer spreading risk 2. CTCs and ctDNA could be used for precision medicine and personalized treatment monitoring	1. Limited available sample for analysis 2. The prognostic significance of CTCs and ctDNA in HNSCC is still unclear.	[108,109,113,114,115,116,117,118,119,120]
Microbiota	PCR of 16S rRNA V1-V4 hypervariable regions in the bacteria	16S rRNA V1-V4 hypervariable regions	The 16S rRNA is amplified by PCR and sequencing by sanger sequencing or NGS	Variation of microbiome correlates with clinical outcomes and epigenetic status.	1. Vague findings between the oral microbiome and HNSCC 2. Need microbiome bank as reference	[125,126,127,128,129,130,131,132,133,134]

CTCs, circulating tumor cells; FDA, Food and Drug Administration; HPV, human papillomavirus; MSI, microsatellite instability; NGS, next-generation sequencing; PCR, polymerase chain reaction; TMB, tumor mutation burden; TME, tumor microenvironment; PD-1, program death-1; PD-L1, program death-ligand 1.

## Abbreviations

APCs	antigen-presenting cells
APOBEC	apolipoprotein-B mRNA editing enzyme catalytic polypeptide-like
Bcl-xL	B cell lymphoma-extra large
bTMB	plasma-based tumor mutational burden
cCSCs	circulating cancer stem-like cells
CPS	combined positive score
CTCs	circulating tumor cells
ctDNA	circulating tumor DNA
CTLA-4	cytotoxic T lymphocyte antigen 4
dMMR	mismatch repair deficiency
EBV	Epstein-Barr virus
EpCAM	Epithelial cell adhesion molecule
FDA	Food and Drug Administration
GEP	gene expression profile
HNSCC	head and neck squamous cell carcinoma
HPV	human papillomavirus
ICIs	immune checkpoint inhibitors
IFN	interferon
IS	immunoscore
LAG-3	lymphocyte-activating gene 3
NIH	National Institutes of Health
Mb	megabase
MSI	microsatellite instability
MSI-H	high microsatellite instability
NK	natural killer
OR	odds ratio
OS	overall survival
PD-1	programmed cell death protein-1
PD-L1	programmed death ligand-1
PFS	progression-free survival
R/M HNSCC	recurrent and metastatic HNSCC
SCC	Squamous cell carcinoma
SOC	standard of care
TCGA	The Cancer Genome Atlas
TIGIT	T cell immunoreceptor with Ig and ITIM domains
TIM-3	T cell immunoglobulin and mucin domain-containing protein 3
TMB	tumor mutational burden
TME	tumor microenvironment
TPS	tumor proportion score
Tregs	regulatory T cells

## References

[1] Haddad R.I., Shin D.M. (2008). Recent advances in head and neck cancer. N. Engl. J. Med..

[2] Lo Nigro C., Denaro N., Merlotti A., Merlano M. (2017). Head and neck cancer: Improving outcomes with a multidisciplinary approach. Cancer Manag. Res..

[3] Blanchard P., Baujat B., Holostenco V., Bourredjem A., Baey C., Bourhis J., Pignon J.P. (2011). Meta-analysis of chemotherapy in head and neck cancer (MACH-NC): A comprehensive analysis by tumour site. Radiother. Oncol..

[4] Forastiere A.A., Zhang Q., Weber R.S., Maor M.H., Goepfert H., Pajak T.F., Morrison W., Glisson B., Trotti A., Ridge J.A. (2013). Long-term results of RTOG 91-11: A comparison of three nonsurgical treatment strategies to preserve the larynx in patients with locally advanced larynx cancer. J. Clin. Oncol..

[5] Vermorken J.B., Mesia R., Rivera F., Remenar E., Kawecki A., Rottey S., Erfan J., Zabolotnyy D., Kienzer H.R., Cupissol D. (2008). Platinum-based chemotherapy plus cetuximab in head and neck cancer. N. Engl. J. Med..

[6] Guigay J., Fayette J., Mesia R., Lafond C., Saada-Bouzid E., Geoffrois L., Martin L., Cupissol D., Capitain O., Castanie H. (2019). TPExtreme randomized trial: TPEx versus Extreme regimen in 1st line recurrent/metastatic head and neck squamous cell carcinoma (R/M HNSCC). J. Clin. Oncol..

[7] Wei S.C., Duffy C.R., Allison J.P. (2018). Fundamental Mechanisms of Immune Checkpoint Blockade Therapy. Cancer Discov..

[8] Robert C., Schachter J., Long G.V., Arance A., Grob J.J., Mortier L., Daud A., Carlino M.S., McNeil C., Lotem M. (2015). Pembrolizumab versus Ipilimumab in Advanced Melanoma. N. Engl. J. Med..

[9] Burtness B., Harrington K.J., Greil R., Soulieres D., Tahara M., de Castro G., Psyrri A., Baste N., Neupane P., Bratland A. (2019). Pembrolizumab alone or with chemotherapy versus cetuximab with chemotherapy for recurrent or metastatic squamous cell carcinoma of the head and neck (KEYNOTE-048): A randomised, open-label, phase 3 study. Lancet.

[10] Gandhi L., Rodriguez-Abreu D., Gadgeel S., Esteban E., Felip E., De Angelis F., Domine M., Clingan P., Hochmair M.J., Powell S.F. (2018). Pembrolizumab plus Chemotherapy in Metastatic Non-Small-Cell Lung Cancer. N. Engl. J. Med..

[11] Ferris R.L., Blumenschein G., Fayette J., Guigay J., Colevas A.D., Licitra L., Harrington K., Kasper S., Vokes E.E., Even C. (2016). Nivolumab for Recurrent Squamous-Cell Carcinoma of the Head and Neck. N. Engl. J. Med..

[12] Harrington K.J., Ferris R.L., Blumenschein G., Colevas A.D., Fayette J., Licitra L., Kasper S., Even C., Vokes E.E., Worden F. (2017). Nivolumab versus standard, single-agent therapy of investigator’s choice in recurrent or metastatic squamous cell carcinoma of the head and neck (CheckMate 141): Health-related quality-of-life results from a randomised, phase 3 trial. Lancet Oncol..

[13] Sharma P., Allison J.P. (2015). The future of immune checkpoint therapy. Science.

[14] Darvin P., Toor S.M., Nair V.S., Elkord E. (2018). Immune checkpoint inhibitors: Recent progress and potential biomarkers. Exp. Mol. Med..

[15] Verma V., Sprave T., Haque W., Simone C.B., Chang J.Y., Welsh J.W., Thomas C.R. (2018). A systematic review of the cost and cost-effectiveness studies of immune checkpoint inhibitors. J. Immunother. Cancer.

[16] Martins F., Sofiya L., Sykiotis G.P., Lamine F., Maillard M., Fraga M., Shabafrouz K., Ribi C., Cairoli A., Guex-Crosier Y. (2019). Adverse effects of immune-checkpoint inhibitors: Epidemiology, management and surveillance. Nat. Rev. Clin. Oncol..

[17] Bajwa R., Cheema A., Khan T., Amirpour A., Paul A., Chaughtai S., Patel S., Patel T., Bramson J., Gupta V. (2019). Adverse Effects of Immune Checkpoint Inhibitors (Programmed Death-1 Inhibitors and Cytotoxic T-Lymphocyte-Associated Protein-4 Inhibitors): Results of a Retrospective Study. J. Clin. Med. Res..

[18] Califf R.M. (2018). Biomarker definitions and their applications. Exp. Biol. Med..

[19] Sawyers C.L. (2008). The cancer biomarker problem. Nature.

[20] Goossens N., Nakagawa S., Sun X., Hoshida Y. (2015). Cancer biomarker discovery and validation. Transl. Cancer Res..

[21] Hsieh J.C., Wang H.M., Wu M.H., Chang K.P., Chang P.H., Liao C.T., Liau C.T. (2019). Review of emerging biomarkers in head and neck squamous cell carcinoma in the era of immunotherapy and targeted therapy. Head Neck.

[22] Chung C.H., Ely K., McGavran L., Varella-Garcia M., Parker J., Parker N., Jarrett C., Carter J., Murphy B.A., Netterville J. (2006). Increased epidermal growth factor receptor gene copy number is associated with poor prognosis in head and neck squamous cell carcinomas. J. Clin. Oncol..

[23] Ang K.K., Berkey B.A., Tu X., Zhang H.Z., Katz R., Hammond E.H., Fu K.K., Milas L. (2002). Impact of epidermal growth factor receptor expression on survival and pattern of relapse in patients with advanced head and neck carcinoma. Cancer Res..

[24] Semrau R., Duerbaum H., Temming S., Huebbers C., Stenner M., Drebber U., Klussmann J.P., Muller R.P., Preuss S.F. (2013). Prognostic impact of human papillomavirus status, survivin, and epidermal growth factor receptor expression on survival in patients treated with radiochemotherapy for very advanced nonresectable oropharyngeal cancer. Head Neck.

[25] Young R.J., Rischin D., Fisher R., McArthur G.A., Fox S.B., Peters L.J., Corry J., Lim A., Waldeck K., Solomon B. (2011). Relationship between epidermal growth factor receptor status, p16(INK4A), and outcome in head and neck squamous cell carcinoma. Cancer Epidemiol. Biomark. Prev..

[26] Rasmussen J.H., Hakansson K., Rasmussen G.B., Vogelius I.R., Friborg J., Fischer B.M., Bentzen S.M., Specht L. (2018). A clinical prognostic model compared to the newly adopted UICC staging in an independent validation cohort of P16 negative/positive head and neck cancer patients. Oral Oncol..

[27] Albers A.E., Qian X., Kaufmann A.M., Coordes A. (2017). Meta analysis: HPV and p16 pattern determines survival in patients with HNSCC and identifies potential new biologic subtype. Sci. Rep..

[28] Zhang W., Edwards A., Fang Z., Flemington E.K., Zhang K. (2016). Integrative Genomics and Transcriptomics Analysis Reveals Potential Mechanisms for Favorable Prognosis of Patients with HPV-Positive Head and Neck Carcinomas. Sci. Rep..

[29] Fakhry C., Westra W.H., Li S., Cmelak A., Ridge J.A., Pinto H., Forastiere A., Gillison M.L. (2008). Improved survival of patients with human papillomavirus-positive head and neck squamous cell carcinoma in a prospective clinical trial. J. Natl. Cancer Inst..

[30] Yu Z., Weinberger P.M., Haffty B.G., Sasaki C., Zerillo C., Joe J., Kowalski D., Dziura J., Camp R.L., Rimm D.L. (2005). Cyclin d1 is a valuable prognostic marker in oropharyngeal squamous cell carcinoma. Clin. Cancer Res..

[31] Zhao Y., Yu D., Li H., Nie P., Zhu Y., Liu S., Zhu M., Fang B. (2014). Cyclin D1 overexpression is associated with poor clinicopathological outcome and survival in oral squamous cell carcinoma in Asian populations: Insights from a meta-analysis. PLoS ONE.

[32] Gallo O., Boddi V., Calzolari A., Simonetti L., Trovati M., Bianchi S. (1996). bcl-2 protein expression correlates with recurrence and survival in early stage head and neck cancer treated by radiotherapy. Clin. Cancer Res..

[33] Lo Muzio L., Falaschini S., Farina A., Rubini C., Pezzetti F., Campisi G., De Rosa G., Capogreco M., Carinci F. (2005). Bcl-2 as prognostic factor in head and neck squamous cell carcinoma. Oncol. Res..

[34] Bauman J.E., Austin M.C., Schmidt R., Kurland B.F., Vaezi A., Hayes D.N., Mendez E., Parvathaneni U., Chai X., Sampath S. (2013). ERCC1 is a prognostic biomarker in locally advanced head and neck cancer: Results from a randomised, phase II trial. Br. J. Cancer.

[35] Hayes M., Lan C., Yan J., Xie Y., Gray T., Amirkhan R.H., Dowell J.E. (2011). ERCC1 expression and outcomes in head and neck cancer treated with concurrent cisplatin and radiation. Anticancer Res..

[36] Rodrigo J.P., Garcia L.A., Ramos S., Lazo P.S., Suarez C. (2000). EMS1 gene amplification correlates with poor prognosis in squamous cell carcinomas of the head and neck. Clin. Cancer Res..

[37] Dubot C., Bernard V., Sablin M.P., Vacher S., Chemlali W., Schnitzler A., Pierron G., Rais K.A., Bessoltane N., Jeannot E. (2018). Comprehensive genomic profiling of head and neck squamous cell carcinoma reveals FGFR1 amplifications and tumour genomic alterations burden as prognostic biomarkers of survival. Eur. J. Cancer.

[38] Ishiguro R., Fujii M., Yamashita T., Tashiro M., Tomita T., Ogawa K., Kameyama K. (2003). CCND1 amplification predicts sensitivity to chemotherapy and chemoradiotherapy in head and neck squamous cell carcinoma. Anticancer Res..

[39] Caponio V.C.A., Troiano G., Adipietro I., Zhurakivska K., Arena C., Mangieri D., Mascitti M., Cirillo N., Lo Muzio L. (2020). Computational analysis of TP53 mutational landscape unveils key prognostic signatures and distinct pathobiological pathways in head and neck squamous cell cancer. Br. J. Cancer.

[40] Nelson M.H., Diven M.A., Huff L.W., Paulos C.M. (2015). Harnessing the Microbiome to Enhance Cancer Immunotherapy. J. Immunol. Res..

[41] Yu T., Guo F., Yu Y., Sun T., Ma D., Han J., Qian Y., Kryczek I., Sun D., Nagarsheth N. (2017). Fusobacterium nucleatum Promotes Chemoresistance to Colorectal Cancer by Modulating Autophagy. Cell.

[42] Oliva M., Spreafico A., Taberna M., Alemany L., Coburn B., Mesia R., Siu L. (2019). Immune biomarkers of response to immune-checkpoint inhibitors in head and neck squamous cell carcinoma. Ann. Oncol..

[43] Pardoll D.M. (2012). The blockade of immune checkpoints in cancer immunotherapy. Nat. Rev. Cancer.

[44] Zhang J., Bu X., Wang H., Zhu Y., Geng Y., Nihira N.T., Tan Y., Ci Y., Wu F., Dai X. (2018). Cyclin D-CDK4 kinase destabilizes PD-L1 via cullin 3-SPOP to control cancer immune surveillance. Nature.

[45] Ngamphaiboon N., Chureemas T., Siripoon T., Arsa L., Trachu N., Jiarpinitnun C., Pattaranutaporn P., Sirachainan E., Larbcharoensub N. (2019). Characteristics and impact of programmed death-ligand 1 expression, CD8+ tumor-infiltrating lymphocytes, and p16 status in head and neck squamous cell carcinoma. Med. Oncol..

[46] Muller T., Braun M., Dietrich D., Aktekin S., Hoft S., Kristiansen G., Goke F., Schrock A., Bragelmann J., Held S.A.E. (2017). PD-L1: A novel prognostic biomarker in head and neck squamous cell carcinoma. Oncotarget.

[47] Okada S., Itoh K., Ishihara S., Shimada J., Kato D., Tsunezuka H., Miyata N., Hirano S., Teramukai S., Inoue M. (2018). Significance of PD-L1 expression in pulmonary metastases from head and neck squamous cell carcinoma. Surg. Oncol..

[48] Hansen A.R., Siu L.L. (2016). PD-L1 Testing in Cancer: Challenges in Companion Diagnostic Development. JAMA Oncol..

[49] Motzer R.J., Tannir N.M., McDermott D.F., Frontera O.A., Melichar B., Choueiri T.K., Plimack E.R., Barthelemy P., Porta C., George S. (2018). Nivolumab plus Ipilimumab versus Sunitinib in Advanced Renal-Cell Carcinoma. N. Engl. J. Med..

[50] Segal N.H., Ou S.I., Balmanoukian A., Fury M.G., Massarelli E., Brahmer J.R., Weiss J., Schoffski P., Antonia S.J., Massard C. (2019). Safety and efficacy of durvalumab in patients with head and neck squamous cell carcinoma: Results from a phase I/II expansion cohort. Eur. J. Cancer.

[51] Cohen E.E.W., Soulieres D., Le Tourneau C., Dinis J., Licitra L., Ahn M.J., Soria A., Machiels J.P., Mach N., Mehra R. (2019). Pembrolizumab versus methotrexate, docetaxel, or cetuximab for recurrent or metastatic head-and-neck squamous cell carcinoma (KEYNOTE-040): A randomised, open-label, phase 3 study. Lancet.

[52] Reck M., Rodriguez-Abreu D., Robinson A.G., Hui R., Csoszi T., Fulop A., Gottfried M., Peled N., Tafreshi A., Cuffe S. (2016). Pembrolizumab versus Chemotherapy for PD-L1-Positive Non-Small-Cell Lung Cancer. N. Engl. J. Med..

[53] Zandberg D.P., Algazi A.P., Jimeno A., Good J.S., Fayette J., Bouganim N., Ready N.E., Clement P.M., Even C., Jang R.W. (2019). Durvalumab for recurrent or metastatic head and neck squamous cell carcinoma: Results from a single-arm, phase II study in patients with >/=25% tumour cell PD-L1 expression who have progressed on platinum-based chemotherapy. Eur. J. Cancer.

[54] Ferris R.L., Blumenschein G., Fayette J., Guigay J., Colevas A.D., Licitra L., Harrington K.J., Kasper S., Vokes E.E., Even C. (2018). Nivolumab vs investigator’s choice in recurrent or metastatic squamous cell carcinoma of the head and neck: 2-year long-term survival update of CheckMate 141 with analyses by tumor PD-L1 expression. Oral Oncol..

[55] Cancer Genome Atlas Network (2015). Comprehensive genomic characterization of head and neck squamous cell carcinomas. Nature.

[56] Lui V.W., Hedberg M.L., Li H., Vangara B.S., Pendleton K., Zeng Y., Lu Y., Zhang Q., Du Y., Gilbert B.R. (2013). Frequent mutation of the PI3K pathway in head and neck cancer defines predictive biomarkers. Cancer Discov..

[57] Leduc C., Adam J., Louvet E., Sourisseau T., Dorvault N., Bernard M., Maingot E., Faivre L., Cassin-Kuo M.S., Boissier E. (2018). TPF induction chemotherapy increases PD-L1 expression in tumour cells and immune cells in head and neck squamous cell carcinoma. ESMO Open.

[58] Cimino-Mathews A., Thompson E., Taube J.M., Ye X., Lu Y., Meeker A., Xu H., Sharma R., Lecksell K., Cornish T.C. (2016). PD-L1 (B7-H1) expression and the immune tumor microenvironment in primary and metastatic breast carcinomas. Hum. Pathol..

[59] Takamori S., Toyokawa G., Okamoto I., Takada K., Kozuma Y., Matsubara T., Haratake N., Akamine T., Katsura M., Mukae N. (2017). Discrepancy in Programmed Cell Death-Ligand 1 Between Primary and Metastatic Non-small Cell Lung Cancer. Anticancer Res..

[60] Jie H.B., Gildener-Leapman N., Li J., Srivastava R.M., Gibson S.P., Whiteside T.L., Ferris R.L. (2013). Intratumoral regulatory T cells upregulate immunosuppressive molecules in head and neck cancer patients. Br. J. Cancer.

[61] Mattox A.K., Lee J., Westra W.H., Pierce R.H., Ghossein R., Faquin W.C., Diefenbach T.J., Morris L.G., Lin D.T., Wirth L.J. (2017). PD-1 Expression in Head and Neck Squamous Cell Carcinomas Derives Primarily from Functionally Anergic CD4(+) TILs in the Presence of PD-L1(+) TAMs. Cancer Res..

[62] Cohen E.E.W., Bell R.B., Bifulco C.B., Burtness B., Gillison M.L., Harrington K.J., Le Q.T., Lee N.Y., Leidner R., Lewis R.L. (2019). The Society for Immunotherapy of Cancer consensus statement on immunotherapy for the treatment of squamous cell carcinoma of the head and neck (HNSCC). J. Immunother. Cancer.

[63] Koppel C., Schwellenbach H., Zielinski D., Eckstein S., Martin-Ortega M., D’Arrigo C., Schildhaus H.U., Ruschoff J., Jasani B. (2018). Optimization and validation of PD-L1 immunohistochemistry staining protocols using the antibody clone 28-8 on different staining platforms. Mod. Pathol..

[64] Sunshine J.C., Nguyen P.L., Kaunitz G.J., Cottrell T.R., Berry S., Esandrio J., Xu H., Ogurtsova A., Bleich K.B., Cornish T.C. (2017). PD-L1 Expression in Melanoma: A Quantitative Immunohistochemical Antibody Comparison. Clin. Cancer Res..

[65] Ionescu D.N., Downes M.R., Christofides A., Tsao M.S. (2018). Harmonization of PD-L1 testing in oncology: A Canadian pathology perspective. Curr. Oncol..

[66] Wang H.C., Chan L.P., Cho S.F. (2019). Targeting the Immune Microenvironment in the Treatment of Head and Neck Squamous Cell Carcinoma. Front. Oncol..

[67] Partlova S., Boucek J., Kloudova K., Lukesova E., Zabrodsky M., Grega M., Fucikova J., Truxova I., Tachezy R., Spisek R. (2015). Distinct patterns of intratumoral immune cell infiltrates in patients with HPV-associated compared to non-virally induced head and neck squamous cell carcinoma. Oncoimmunology.

[68] Wang J., Sun H., Zeng Q., Guo X.J., Wang H., Liu H.H., Dong Z.Y. (2019). HPV-positive status associated with inflamed immune microenvironment and improved response to anti-PD-1 therapy in head and neck squamous cell carcinoma. Sci. Rep..

[69] Lechner A., Schlosser H.A., Thelen M., Wennhold K., Rothschild S.I., Gilles R., Quaas A., Siefer O.G., Huebbers C.U., Cukuroglu E. (2019). Tumor-associated B cells and humoral immune response in head and neck squamous cell carcinoma. Oncoimmunology.

[70] Kim H.R., Ha S.J., Hong M.H., Heo S.J., Koh Y.W., Choi E.C., Kim E.K., Pyo K.H., Jung I., Seo D. (2016). PD-L1 expression on immune cells, but not on tumor cells, is a favorable prognostic factor for head and neck cancer patients. Sci. Rep..

[71] Mandal R., Senbabaoglu Y., Desrichard A., Havel J.J., Dalin M.G., Riaz N., Lee K.W., Ganly I., Hakimi A.A., Chan T.A. (2016). The head and neck cancer immune landscape and its immunotherapeutic implications. JCI Insight.

[72] Chow L.Q.M., Haddad R., Gupta S., Mahipal A., Mehra R., Tahara M., Berger R., Eder J.P., Burtness B., Lee S.H. (2016). Antitumor Activity of Pembrolizumab in Biomarker-Unselected Patients With Recurrent and/or Metastatic Head and Neck Squamous Cell Carcinoma: Results From the Phase Ib KEYNOTE-012 Expansion Cohort. J. Clin. Oncol..

[73] Bauml J., Seiwert T.Y., Pfister D.G., Worden F., Liu S.V., Gilbert J., Saba N.F., Weiss J., Wirth L., Sukari A. (2017). Pembrolizumab for Platinum- and Cetuximab-Refractory Head and Neck Cancer: Results From a Single-Arm, Phase II Study. J. Clin. Oncol..

[74] Colevas A.D., Bahleda R., Braiteh F., Balmanoukian A., Brana I., Chau N.G., Sarkar I., Molinero L., Grossman W., Kabbinavar F. (2018). Safety and clinical activity of atezolizumab in head and neck cancer: Results from a phase I trial. Ann. Oncol..

[75] Henderson S., Chakravarthy A., Su X., Boshoff C., Fenton T.R. (2014). APOBEC-mediated cytosine deamination links PIK3CA helical domain mutations to human papillomavirus-driven tumor development. Cell Rep..

[76] Cannataro V.L., Gaffney S.G., Sasaki T., Issaeva N., Grewal N.K.S., Grandis J.R., Yarbrough W.G., Burtness B., Anderson K.S., Townsend J.P. (2019). APOBEC-induced mutations and their cancer effect size in head and neck squamous cell carcinoma. Oncogene.

[77] Faden D.L., Thomas S., Cantalupo P.G., Agrawal N., Myers J., DeRisi J. (2017). Multi-modality analysis supports APOBEC as a major source of mutations in head and neck squamous cell carcinoma. Oral Oncol..

[78] Faden D.L., Ding F., Lin Y., Zhai S., Kuo F., Chan T.A., Morris L.G., Ferris R.L. (2019). APOBEC mutagenesis is tightly linked to the immune landscape and immunotherapy biomarkers in head and neck squamous cell carcinoma. Oral Oncol..

[79] McGranahan N., Furness A.J., Rosenthal R., Ramskov S., Lyngaa R., Saini S.K., Jamal-Hanjani M., Wilson G.A., Birkbak N.J., Hiley C.T. (2016). Clonal neoantigens elicit T cell immunoreactivity and sensitivity to immune checkpoint blockade. Science.

[80] Yarchoan M., Hopkins A., Jaffee E.M. (2017). Tumor Mutational Burden and Response Rate to PD-1 Inhibition. N. Engl. J. Med..

[81] Hellmann M.D., Ciuleanu T.E., Pluzanski A., Lee J.S., Otterson G.A., Audigier-Valette C., Minenza E., Linardou H., Burgers S., Salman P. (2018). Nivolumab plus Ipilimumab in Lung Cancer with a High Tumor Mutational Burden. N. Engl. J. Med..

[82] Seiwert T.Y., Haddad R., Bauml J., Weiss J., Pfister D.G., Gupta S., Mehra R., Gluck I., Kang H., Worden F. (2018). Abstract LB-339: Biomarkers predictive of response to pembrolizumab in head and neck cancer (HNSCC). Cancer Res..

[83] Cristescu R., Mogg R., Ayers M., Albright A., Murphy E., Yearley J., Sher X., Liu X.Q., Lu H., Nebozhyn M. (2018). Pan-tumor genomic biomarkers for PD-1 checkpoint blockade-based immunotherapy. Science.

[84] Hanna G.J., Lizotte P., Cavanaugh M., Kuo F.C., Shivdasani P., Frieden A., Chau N.G., Schoenfeld J.D., Lorch J.H., Uppaluri R. (2018). Frameshift events predict anti-PD-1/L1 response in head and neck cancer. JCI Insight.

[85] Li W., Wildsmith S., Ye J., Si H., Morsli N., He P., Shetty J., Yovine A.J., Holoweckyj N., Raja R. (2020). Plasma-based tumor mutational burden (bTMB) as predictor for survival in phase III EAGLE study: Durvalumab (D) ± tremelimumab (T) versus chemotherapy (CT) in recurrent/metastatic head and neck squamous cell carcinoma (R/M HNSCC) after platinum failure. J. Clin. Oncol..

[86] Hladikova K., Koucky V., Boucek J., Laco J., Grega M., Hodek M., Zabrodsky M., Vosmik M., Rozkosova K., Vosmikova H. (2019). Tumor-infiltrating B cells affect the progression of oropharyngeal squamous cell carcinoma via cell-to-cell interactions with CD8(+) T cells. J. Immunother. Cancer.

[87] Galon J., Fox B.A., Bifulco C.B., Masucci G., Rau T., Botti G., Marincola F.M., Ciliberto G., Pages F., Ascierto P.A. (2016). Immunoscore and Immunoprofiling in cancer: An update from the melanoma and immunotherapy bridge 2015. J. Transl. Med..

[88] Galon J., Costes A., Sanchez-Cabo F., Kirilovsky A., Mlecnik B., Lagorce-Pages C., Tosolini M., Camus M., Berger A., Wind P. (2006). Type, density, and location of immune cells within human colorectal tumors predict clinical outcome. Science.

[89] Donnem T., Hald S.M., Paulsen E.E., Richardsen E., Al-Saad S., Kilvaer T.K., Brustugun O.T., Helland A., Lund-Iversen M., Poehl M. (2015). Stromal CD8+ T-cell Density-A Promising Supplement to TNM Staging in Non-Small Cell Lung Cancer. Clin. Cancer Res..

[90] Zhang X.M., Song L.J., Shen J., Yue H., Han Y.Q., Yang C.L., Liu S.Y., Deng J.W., Jiang Y., Fu G.H. (2018). Prognostic and predictive values of immune infiltrate in patients with head and neck squamous cell carcinoma. Hum. Pathol..

[91] Lechner A., Schlosser H., Rothschild S.I., Thelen M., Reuter S., Zentis P., Shimabukuro-Vornhagen A., Theurich S., Wennhold K., Garcia-Marquez M. (2017). Characterization of tumor-associated T-lymphocyte subsets and immune checkpoint molecules in head and neck squamous cell carcinoma. Oncotarget.

[92] Anderson A.C., Joller N., Kuchroo V.K. (2016). Lag-3, Tim-3, and TIGIT: Co-inhibitory Receptors with Specialized Functions in Immune Regulation. Immunity.

[93] Koyama S., Akbay E.A., Li Y.Y., Herter-Sprie G.S., Buczkowski K.A., Richards W.G., Gandhi L., Redig A.J., Rodig S.J., Asahina H. (2016). Adaptive resistance to therapeutic PD-1 blockade is associated with upregulation of alternative immune checkpoints. Nat. Commun..

[94] Thommen D.S., Schreiner J., Muller P., Herzig P., Roller A., Belousov A., Umana P., Pisa P., Klein C., Bacac M. (2015). Progression of Lung Cancer Is Associated with Increased Dysfunction of T Cells Defined by Coexpression of Multiple Inhibitory Receptors. Cancer Immunol. Res..

[95] Chauvin J.M., Pagliano O., Fourcade J., Sun Z., Wang H., Sander C., Kirkwood J.M., Chen T.H., Maurer M., Korman A.J. (2015). TIGIT and PD-1 impair tumor antigen-specific CD8(+) T cells in melanoma patients. J. Clin. Investig..

[96] Jenkins R.W., Barbie D.A., Flaherty K.T. (2018). Mechanisms of resistance to immune checkpoint inhibitors. Br. J. Cancer.

[97] Haddad R., Concha-Benavente F., Blumenschein G., Fayette J., Guigay J., Colevas A.D., Licitra L., Kasper S., Vokes E.E., Worden F. (2019). Nivolumab treatment beyond RECIST-defined progression in recurrent or metastatic squamous cell carcinoma of the head and neck in CheckMate 141: A subgroup analysis of a randomized phase 3 clinical trial. Cancer.

[98] Jamieson N.B., Maker A.V. (2017). Gene-expression profiling to predict responsiveness to immunotherapy. Cancer Gene Ther..

[99] Prat A., Navarro A., Pare L., Reguart N., Galvan P., Pascual T., Martinez A., Nuciforo P., Comerma L., Alos L. (2017). Immune-Related Gene Expression Profiling After PD-1 Blockade in Non-Small Cell Lung Carcinoma, Head and Neck Squamous Cell Carcinoma, and Melanoma. Cancer Res..

[100] Seiwert T.Y., Burtness B., Mehra R., Weiss J., Berger R., Eder J.P., Heath K., McClanahan T., Lunceford J., Gause C. (2016). Safety and clinical activity of pembrolizumab for treatment of recurrent or metastatic squamous cell carcinoma of the head and neck (KEYNOTE-012): An open-label, multicentre, phase 1b trial. Lancet Oncol..

[101] Hernandez C.P., Morrow K., Velasco C., Wyczechowska D.D., Naura A.S., Rodriguez P.C. (2013). Effects of cigarette smoke extract on primary activated T cells. Cell. Immunol..

[102] Desrichard A., Kuo F., Chowell D., Lee K.W., Riaz N., Wong R.J., Chan T.A., Morris L.G.T. (2018). Tobacco Smoking-Associated Alterations in the Immune Microenvironment of Squamous Cell Carcinomas. J. Natl. Cancer Inst..

[103] Gildener-Leapman N., Ferris R.L., Bauman J.E. (2013). Promising systemic immunotherapies in head and neck squamous cell carcinoma. Oral Oncol..

[104] Le D.T., Durham J.N., Smith K.N., Wang H., Bartlett B.R., Aulakh L.K., Lu S., Kemberling H., Wilt C., Luber B.S. (2017). Mismatch repair deficiency predicts response of solid tumors to PD-1 blockade. Science.

[105] Le D.T., Uram J.N., Wang H., Bartlett B.R., Kemberling H., Eyring A.D., Skora A.D., Luber B.S., Azad N.S., Laheru D. (2015). PD-1 Blockade in Tumors with Mismatch-Repair Deficiency. N. Engl. J. Med..

[106] Martens A., Wistuba-Hamprecht K., Foppen M.G., Yuan J., Postow M.A., Wong P., Romano E., Khammari A., Dreno B., Capone M. (2016). Baseline Peripheral Blood Biomarkers Associated with Clinical Outcome of Advanced Melanoma Patients Treated with Ipilimumab. Clin. Cancer Res..

[107] Tardy M.P., Di Mauro I., Ebran N., Refae S., Bozec A., Benezery K., Peyrade F., Guigay J., Sudaka-Bahadoran A., Badoual C. (2018). Microsatellite instability associated with durable complete response to PD-L1 inhibitor in head and neck squamous cell carcinoma. Oral Oncol..

[108] Luchini C., Bibeau F., Ligtenberg M.J.L., Singh N., Nottegar A., Bosse T., Miller R., Riaz N., Douillard J.Y., Andre F. (2019). ESMO recommendations on microsatellite instability testing for immunotherapy in cancer, and its relationship with PD-1/PD-L1 expression and tumour mutational burden: A systematic review-based approach. Ann. Oncol..

[109] Zavridou M., Mastoraki S., Strati A., Koutsodontis G., Klinakis A., Psyrri A., Lianidou E. (2020). Direct comparison of size-dependent versus EpCAM-dependent CTC enrichment at the gene expression and DNA methylation level in head and neck squamous cell carcinoma. Sci. Rep..

[110] Tada H., Takahashi H., Kuwabara-Yokobori Y., Shino M., Chikamatsu K. (2020). Molecular profiling of circulating tumor cells predicts clinical outcome in head and neck squamous cell carcinoma. Oral Oncol..

[111] Economopoulou P., Kotsantis I., Kyrodimos E., Lianidou E.S., Psyrri A. (2017). Liquid biopsy: An emerging prognostic and predictive tool in Head and Neck Squamous Cell Carcinoma (HNSCC). Focus on Circulating Tumor Cells (CTCs). Oral Oncol..

[112] Misawa K., Imai A., Matsui H., Kanai A., Misawa Y., Mochizuki D., Mima M., Yamada S., Kurokawa T., Nakagawa T. (2020). Identification of novel methylation markers in HPV-associated oropharyngeal cancer: Genome-wide discovery, tissue verification and validation testing in ctDNA. Oncogene.

[113] Bratman S.V., Yang S.C., Iafolla M.A., Liu Z., Hansen A.R., Bedard P.L., Lheureux S., Spreafico A., Razak A.A., Shchegrova S. (2020). Personalized circulating tumor DNA analysis as a predictive biomarker in solid tumor patients treated with pembrolizumab. Nat. Cancer.

[114] Xun Y., Cao Q., Zhang J., Guan B., Wang M. (2020). Clinicopathological and prognostic significance of circulating tumor cells in head and neck squamous cell carcinoma: A systematic review and meta-analysis. Oral Oncol..

[115] Liu K., Chen N., Wei J., Ma L., Yang S., Zhang X. (2020). Clinical significance of circulating tumor cells in patients with locally advanced head and neck squamous cell carcinoma. Oncol. Rep..

[116] Alix-Panabieres C. (2020). The future of liquid biopsy. Nature.

[117] Tinhofer I., Konschak R., Stromberger C., Raguse J.D., Dreyer J.H., Johrens K., Keilholz U., Budach V. (2014). Detection of circulating tumor cells for prediction of recurrence after adjuvant chemoradiation in locally advanced squamous cell carcinoma of the head and neck. Ann. Oncol..

[118] Grobe A., Blessmann M., Hanken H., Friedrich R.E., Schon G., Wikner J., Effenberger K.E., Kluwe L., Heiland M., Pantel K. (2014). Prognostic relevance of circulating tumor cells in blood and disseminated tumor cells in bone marrow of patients with squamous cell carcinoma of the oral cavity. Clin. Cancer Res..

[119] Wang H.M., Wu M.H., Chang P.H., Lin H.C., Liao C.D., Wu S.M., Hung T.M., Lin C.Y., Chang T.C., Tzu-Tsen Y. (2019). The change in circulating tumor cells before and during concurrent chemoradiotherapy is associated with survival in patients with locally advanced head and neck cancer. Head Neck.

[120] Strati A., Koutsodontis G., Papaxoinis G., Angelidis I., Zavridou M., Economopoulou P., Kotsantis I., Avgeris M., Mazel M., Perisanidis C. (2017). Prognostic significance of PD-L1 expression on circulating tumor cells in patients with head and neck squamous cell carcinoma. Ann. Oncol..

[121] Rao V.U., Arakeri G., Subash A., Bagadia R.K., Thakur S., Kudpaje A.S., Nayar R., Patil S., Paiva Fonseca F., Gomez R.S. (2020). Circulating tumour cells in head and neck cancers: Biological insights. J. Oral Pathol. Med..

[122] Chang P.H., Wu M.H., Liu S.Y., Wang H.M., Huang W.K., Liao C.T., Yen T.C., Ng S.H., Chen J.S., Lin Y.C. (2019). The Prognostic Roles of Pretreatment Circulating Tumor Cells, Circulating Cancer Stem-Like Cells, and Programmed Cell Death-1 Expression on Peripheral Lymphocytes in Patients with Initially Unresectable, Recurrent or Metastatic Head and Neck Cancer: An Exploratory Study of Three Biomarkers in One-time Blood Drawing. Cancers.

[123] Guibert N., Delaunay M., Lusque A., Boubekeur N., Rouquette I., Clermont E., Mourlanette J., Gouin S., Dormoy I., Favre G. (2018). PD-L1 expression in circulating tumor cells of advanced non-small cell lung cancer patients treated with nivolumab. Lung Cancer.

[124] Nicolazzo C., Raimondi C., Mancini M., Caponnetto S., Gradilone A., Gandini O., Mastromartino M., Del Bene G., Prete A., Longo F. (2016). Monitoring PD-L1 positive circulating tumor cells in non-small cell lung cancer patients treated with the PD-1 inhibitor Nivolumab. Sci. Rep..

[125] Dhar M., Wong J., Che J., Matsumoto M., Grogan T., Elashoff D., Garon E.B., Goldman J.W., Sollier Christen E., Di Carlo D. (2018). Evaluation of PD-L1 expression on vortex-isolated circulating tumor cells in metastatic lung cancer. Sci. Rep..

[126] Roy S., Trinchieri G. (2017). Microbiota: A key orchestrator of cancer therapy. Nat. Rev. Cancer.

[127] Greenhill C. (2014). Gut microbiota: Anti-cancer therapies affected by gut microbiota. Nat. Rev. Gastroenterol. Hepatol..

[128] Kroemer G., Zitvogel L. (2018). Cancer immunotherapy in 2017: The breakthrough of the microbiota. Nat. Rev. Immunol..

[129] Brandi G., Frega G. (2019). Microbiota: Overview and Implication in Immunotherapy-Based Cancer Treatments. Int. J. Mol. Sci..

[130] Gopalakrishnan V., Spencer C.N., Nezi L., Reuben A., Andrews M.C., Karpinets T.V., Prieto P.A., Vicente D., Hoffman K., Wei S.C. (2018). Gut microbiome modulates response to anti-PD-1 immunotherapy in melanoma patients. Science.

[131] Le Bars P., Matamoros S., Montassier E., Le Vacon F., Potel G., Soueidan A., Jordana F., de La Cochetiere M.F. (2017). The oral cavity microbiota: Between health, oral disease, and cancers of the aerodigestive tract. Can. J. Microbiol..

[132] Pushalkar S., Ji X., Li Y., Estilo C., Yegnanarayana R., Singh B., Li X., Saxena D. (2012). Comparison of oral microbiota in tumor and non-tumor tissues of patients with oral squamous cell carcinoma. BMC Microbiol..

[133] Hooper S.J., Wilson M.J., Crean S.J. (2009). Exploring the link between microorganisms and oral cancer: A systematic review of the literature. Head Neck.

[134] Shin J.M., Luo T., Kamarajan P., Fenno J.C., Rickard A.H., Kapila Y.L. (2017). Microbial Communities Associated with Primary and Metastatic Head and Neck Squamous Cell Carcinoma—A High Fusobacterial and Low Streptococcal Signature. Sci. Rep..

[135] Ferris R.L., Blumenschein G., Harrington K., Fayette J., Guigay J., Colevas A.D., Licitra L., Vokes E., Gillison M., Even C. (2017). Abstract CT022: Evaluation of oral microbiome profiling as a response biomarker in squamous cell carcinoma of the head and neck: Analyses from CheckMate 141. Cancer Res..

[136] Ho B.X., Pek N.M.Q., Soh B.-S. (2018). Disease modeling using 3D organoids derived from human induced pluripotent stem cells. Int. J. Mol. Sci..

[137] Dutta D., Heo I., Clevers H. (2017). Disease Modeling in Stem Cell-Derived 3D Organoid Systems. Trends Mol. Med..

[138] Neal J.T., Li X., Zhu J., Giangarra V., Grzeskowiak C.L., Ju J., Liu I.H., Chiou S.H., Salahudeen A.A., Smith A.R. (2018). Organoid Modeling of the Tumor Immune Microenvironment. Cell.

[139] Yuki K., Cheng N., Nakano M., Kuo C.J. (2020). Organoid Models of Tumor Immunology. Trends Immunol..

[140] Scognamiglio G., De Chiara A., Parafioriti A., Armiraglio E., Fazioli F., Gallo M., Aversa L., Camerlingo R., Cacciatore F., Colella G. (2019). Patient-derived organoids as a potential model to predict response to PD-1/PD-L1 checkpoint inhibitors. Br. J. Cancer.

[141] Driehuis E., Kolders S., Spelier S., Lõhmussaar K., Willems S., Devriese L., de Bree R., de Ruiter E., Korving J., Begthel H. (2019). Oral mucosal organoids as a potential platform for personalized cancer therapy. Cancer Discov..

[142] Lee J.S., Ruppin E. (2019). Multiomics Prediction of Response Rates to Therapies to Inhibit Programmed Cell Death 1 and Programmed Cell Death 1 Ligand 1. JAMA Oncol..

[143] Trebeschi S., Drago S.G., Birkbak N.J., Kurilova I., Calin A.M., Pizzi A.D., Lalezari F., Lambregts D.M.J., Rohaan M.W., Parmar C. (2019). Predicting response to cancer immunotherapy using noninvasive radiomic biomarkers. Ann. Oncol..

[144] Yu K.H., Beam A.L., Kohane I.S. (2018). Artificial intelligence in healthcare. Nat. Biomed. Eng..

